# The Complexity of a Dengue Vaccine: A Review of the Human Antibody Response

**DOI:** 10.1371/journal.pntd.0003749

**Published:** 2015-06-11

**Authors:** Jacky Flipse, Jolanda M. Smit

**Affiliations:** Department of Medical Microbiology, University Medical Center Groningen, University of Groningen, Groningen, The Netherlands; Oxford University Clinical Research Unit, VIETNAM

## Abstract

Dengue is the most prevalent mosquito-borne viral disease worldwide. Yet, there are no vaccines or specific antivirals available to prevent or treat the disease. Several dengue vaccines are currently in clinical or preclinical stages. The most advanced vaccine is the chimeric tetravalent CYD-TDV vaccine of Sanofi Pasteur. This vaccine has recently cleared Phase III, and efficacy results have been published. Excellent tetravalent seroconversion was seen, yet the protective efficacy against infection was surprisingly low. Here, we will describe the complicating factors involved in the generation of a safe and efficacious dengue vaccine. Furthermore, we will discuss the human antibody responses during infection, including the epitopes targeted in humans. Also, we will discuss the current understanding of the assays used to evaluate antibody response. We hope this review will aid future dengue vaccine development as well as fundamental research related to the phenomenon of antibody-dependent enhancement of dengue virus infection.

## Introduction

**Fig 1:**
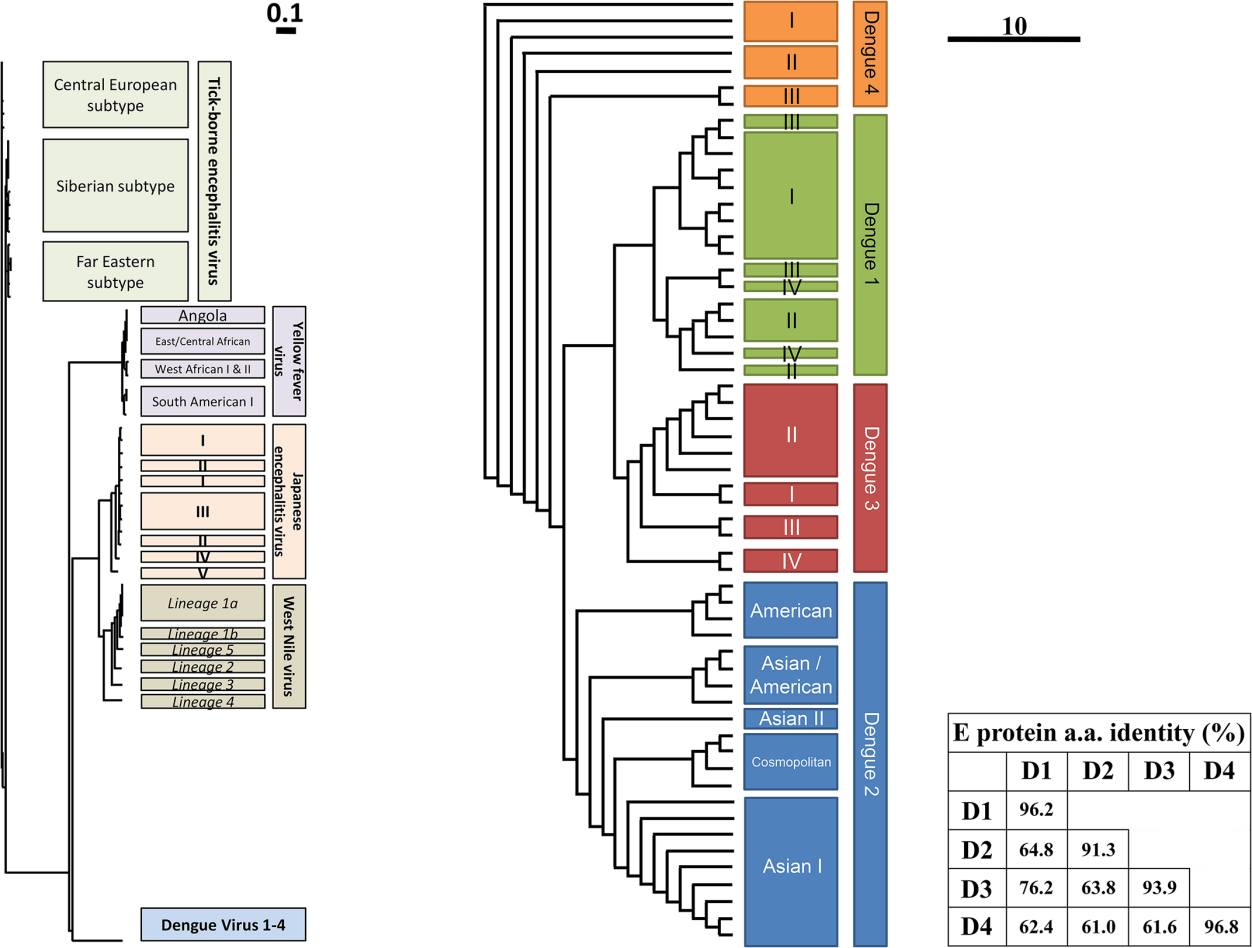
Close relationship between several flaviviruses (left) and within the species of dengue virus (right). The phylogenetic tree is based on the amino acid sequence of the envelope glycoproteins. The methodology and National Center for Biotechnology Information (NCBI) IDs of all used genotypes for the flaviviruses and dengue viruses are provided in S1 Dataset. The table denominates the percentage of consensus between the serotypes based on the envelope amino acid sequences. Sequence identities were calculated using the Sequence Identity and Similarity (SIAS) calculator (http://imed.med.ucm.es/Tools/sias.html). Scale bar of 0.1 (flaviviruses) or 10 (dengue virus) denotes 0.1 or 10 (silent) substitutions per amino acid for the flavivirus and dengue sequences, respectively.

The genus *Flavivirus* of the family *Flaviviridae* comprises over 50 closely related viruses, including dengue virus (DENV), Japanese encephalitis virus (JEV), yellow fever virus (YFV), tick-borne encephalitis virus (TBEV), and West Nile virus (WNV) (Fig 1). Flaviviruses are arthropod-borne pathogens, and transmission occurs by ticks (TBEV) or mosquitoes (e.g., JEV and DENV). Flaviviruses are present worldwide, ranging from the tropics (JEV and DENV), to moderate climates (DENV and WNV), to near-arctic climate (TBEV) [1].

Infection with a flavivirus can cause a wide range of clinically overt symptoms [1,2], potentially resulting in death. For example, JEV is the leading cause of viral encephalitis in Asia, with a 30%–40% case fatality rate [2]. Dengue is the most common arthropod-borne viral infection occurring worldwide, with an estimated 360 million infections and 96 million symptomatic cases in 2010 [3]. On average, 500,000–1 million individuals develop severe disease, including hemorrhage and plasma leakage, resulting in 25,000 deaths [4].

Currently, there are vaccines available for YFV, TBEV, and JEV. Yet, there is no vaccine available for the closely related DENV [5]. This is in part due to the existence of four genetically and antigenically distinct DENV serotypes (Fig 1). There is approximately 40% divergence between the amino acid sequences of the serotypes (Fig 1) [6,7] and up to ≤9% mismatch within a serotype (Fig 1) [8]. The diversity of the genotypes of JEV, WNV, and TBEV is much less, with ≤4.1%, ≤2%, and ≤5.6% difference, respectively [9,10]; therefore, no distinct serotypes exist.

Another factor for the complexity of the DENV vaccine lies in the severity of disease. All four DENV serotypes can cause symptoms ranging from acute febrile illness to severe manifestations as hemorrhage or organ impairment. Severe disease is most often seen during secondary, heterotypic reinfections [11,12]. The incidence of severe disease during secondary, heterologous infection relative to primary infection can be 20-fold to 80-fold higher [12–15]. The observation that disease can be more severe during secondary infections severely hampered the development of a vaccine, as it implies the need to simultaneously induce immunity to all four existing DENV serotypes over a prolonged period [16,17].

Multiple vaccine formulations are currently being tested in preclinical and clinical stages, and these have been reviewed before [18]. Here, we will focus on the Sanofi Pasteur live attenuated vaccine since this is the most advanced vaccine with known efficacy results. The results of the trials will be reviewed and discussed within the context of the host immune response and the assays used to understand and evaluate both the vaccine and the host immune response.

## Sanofi Trials

Sanofi Pasteur developed a tetravalent chimeric YFV/DENV vaccine (CYD-TDV). The vaccine was based on the backbone of the attenuated YFV strain 17D in which the structural genes encoding for the premembrane (prM) and envelope (E) proteins of YFV were replaced with those of DENV [19]. YFV/DENV chimeric viruses were made from all four DENV serotypes. The resulting viruses thus have the attenuated replication machinery of YFV and the outer structure of a DENV serotype. Hence, the vaccine induces CD4^+^ T cell and antibody responses against the DENV structural proteins and CD8^+^ T cell responses against the YFV nonstructural (NS) proteins [20–22]. Preclinical in vitro assays showed genomic stability and no toxicity (reviewed in [19]) and induction of antiviral responses in human dendritic cells [23].

Subsequently, clinical studies were performed using a three-dose regimen containing 10^5^ CCID_50_ of each YFV/DENV chimeric virus. The Phase I and II trials showed that the vaccine is safe and tolerable in humans [19,24], which was the primary end point. Additionally, the authors of the Phase II trials also determined the seroconversion and the efficacy against virologically confirmed DENV. In one study, excellent tetravalent seroconversion against DENV was noted, as 95%–100% of the individuals seroconverted [25]. Yet, in the same study, the efficacy was surprisingly low, being 30%, whilst another study reported near 64% efficacy (Table 1). These Phase II trials were conducted with relatively low numbers of participants. Next, large Phase III trials were conducted in Asia and Latin America to determine the efficacy of the vaccine. However, the recent reports of these trials were quite enigmatic. The Phase III studies in Southeast Asia and South America reported an efficacy range of 51.1%–79% and 31.3%–77.5%, respectively. Overall, the vaccine was shown to be efficacious, as the 95% CI was higher than 25% (primary end point). It should be noted, however, that the reported efficacies varied per country and per study. Additionally, when the serotype specific efficacy was calculated, the lowest efficacy was consistently seen for DENV2 (Table 1).

**Table 1:** An overview of the results from the CYD-TDV vaccine trials.

Reference	Age Range (years)	Area	Efficacy			Baseline Immunity (%)		Effect of Baseline Immunity
			Post Third Dose: Overall (95% CI).Serotype-Specific, DENV1/2/3/4	Hosp.	DHF	DENV	Flavi	
[25]	4–11	Thailand	30.2%(-13.4 to 56.6),55.6/9.2/75.3/100 ^†^			69.9	91	
[26]	9–16	Honduras, Colombia, Mexico, and Puerto Rico	63.9% (1.5 to 87.4). ^†^			76	79.3	Flavi^+^ > naïve (tetravalent: 97.6% versus 77.9%)
[27]	4–11	Indonesia, Malaysia, Philippine, Thailand, and Vietnam	56.5% (43.8 to 66.4).50/35/78.4/75.3	67.2	80.8	67.6	78.2	DENV^+^ > DENV^**-**^ (efficacy: 74.3% versus 35.5%)
[28]	9–16	Colombia, Brazil, Mexico, Puerto Rico, and Honduras	60.8% (52.0 to 68.0).50.3/42.3/74/77.7	80.3	90.0	79.4		DENV^+^ > DENV^**-**^ (efficacy: 83.7% versus 43.2%)

95% CI, 95% confidence interval; Hosp., hospitalization; DHF, dengue hemorrhagic fever.

^**†**^ Study was a Phase II clinical trial, with a relatively low number of participants.

Strikingly, the vaccine cohort had significantly lower incidence of dengue hemorrhagic fever (80%–90% efficacy) and hospitalization (67%–80% efficacy) [27,28]. Baseline immunity seems to be beneficial in terms of developing tetravalent seroconversion and overall efficacy against symptomatic DENV (Table 1).

While the protection against hemorrhagic fever is encouraging, these trials also taught us that seroconversion alone does not predict protective efficacy. Clearly, more research is required to identify the correlate of protection [29]. Furthermore, it showed us that we need to have a better understanding of the immune response to DENV infection. Hence, below we will discuss what is known about the function of T and B cells in immunity against DENV. Most attention has been directed towards the role of antibodies in immunity against DENV, and therefore, these will be the primary focus of this review.

## Human Immune Response and Disease

After a primary DENV infection, individuals are protected against disease upon reinfection with the homologous serotype. Cross-protection against other serotypes is limited and exists only for 1–2 months post–primary infection, while disease severity was found to be alleviated for 2–9 months thereafter [30,31]. Recent information suggests that cross-protection against severe disease lasts up to 2 years [32–35]. Intriguingly, after the cross-protective period, individuals are at risk of developing more severe dengue upon secondary infection with a heterotypic serotype. Moreover, the chance to develop severe disease increases with the time between the primary and the secondary infection [33,34].

The increased chance of severe disease can be explained by original antigenic sin, a phenomenon in which the human immune system preferentially activates memory T and B cells against the original antigen rather than instructing naïve T and B cells against the current antigen [36,37]. Indeed, it was found that upon a secondary heterotypic DENV infection, the acute T cell response is mostly directed towards the previous infecting serotype [38,39]. Over time, the T cells against conserved, cross-reactive epitopes are preferentially expanded, resulting in a DENV-broad [20,38,40] and potentially flavivirus-broad response [39,41]. As for B cells, a predominant monotypic response with high avidity against the infecting serotype is observed 6–9 days after disease onset [42,43]. Yet, within 6 months of infection, a broad cross-reactive B cell repertoire is seen [43]. Indeed, cross-reactive B cells are predominantly present at the time of secondary infection [42]. These cells have been speculated to contribute to enhanced severity of dengue disease severity [44] (discussed below). After a secondary heterotypic infection, stable populations of DENV-broad cross-reactive B cells are seen [42,43], and these cells secrete high levels of high-avidity antibodies [42,45,46].

Antibodies are suggested to be more important than T cells in triggering the onset of severe disease. This was suggested because infants born to dengue immune mothers were noted to have a higher risk for severe disease development during primary infection [47]. Halstead and others found that waning antibody titers can enhance DENV infectivity in vitro and in vivo [48–50] and developed the theory of antibody-dependent enhancement (ADE) of disease [48,51]. During ADE, the pre-existing cross-reactive antibodies bind to the newly infecting DENV serotype and specifically target the immune complexes to Fc-receptor-expressing cells, cells that are highly permissive to DENV. The high viral burden triggers the immune system, which at the end is responsible for the onset of severe signs like plasma leakage [51–53].

Thus, in case of dengue, antibodies have a paradoxical role: antibodies induced during a primary infection are believed to confer lifelong protection against the infecting serotype, whereas upon reinfection with another DENV serotype, these antibodies can contribute to severe disease development. Hence, we wished to gather information on the human antibody epitopes and their relative contributions to the human antibody repertoire after DENV vaccination and infection. Although we primarily focus on antibody epitopes, we also included a brief description of the role of T cells in connection with the CYD vaccine.

### Human Antibody Responses

We first reviewed the antibody responses in the sera of primary and secondary DENV cases (S1 Table). The majority of antibodies are raised against the E protein, and a small fraction target the prM and the NS proteins. This is not very surprising as E and prM are exposed on the viral surface and soluble NS1 is secreted by infected cells [54]. The higher fraction of E protein antibodies suggests that the human antibody response predominantly targets DENV particles (structural proteins) rather than NS1-positive cells, i.e., infected cells or cells having bound soluble NS1 [55,56]. Interestingly, we see that during secondary infection the antibody repertoire broadens as higher responses against the prM and NS1 proteins are seen. This implies that antibodies against E, prM, and NS1 are differentially induced between primary and secondary infection (discussed further below). A detailed insight in the specific antibody repertoire may therefore help us to better understand the contribution of distinct epitopes to infection neutralization.

Indeed, several elegant studies have used immortalized B cells from human blood samples to generate monoclonal antibodies of these cultures. Unfortunately, the studies conducted so far show considerable variability in numbers and epitopes of antibodies isolated from individual patients (S2 Table). This is likely due to differences in donor backgrounds and immortalizing method used. Therefore, we next focused on those studies in which primary and secondary antibody responses or acute and convalescent samples are compared (Table 2). Even then, the results are highly variable: e.g., the prM response strongly expands in two studies but decreased in one study. The latter study also showed a stable E response between primary and secondary responses, while the others reported a reduction thereof. Yet, when we looked at both sera and monoclonals (S1 and S2 Tables), overall, the E antibodies are dominant during the primary response. The results for secondary responses are more variable (Table 2), but in sera prM and NS antibodies are particularly detected in secondary cases (S1 Table).

**Table 2:** Temporal evaluation of human B cell-derived monoclonal antibodies against DENV.

Reference	Stage	# Donors	# mAbs	NS1	prM	E	As % of Total E	
							EDI/DII	EDIII
[57]	1st, convalescent	3	49	8.0%	5.7%	80.5%	72.6%	27.4%
	2nd, convalescent	2	29	0.0%	2.6%	94.8%	75.0%	25.0%
[58]	1st	6	28	n.d.	14.3%	85.7%	82.8%	17.2%
	2nd	6	9		44.4%	55.6%		
[59]	2nd acute	4	121	3.3%	6.6%	81.8%		
	2nd, convalescent	5	15	53.3%	13.3%	13.3%		

To generate the monoclonal antibodies (mAbs) listed in this table, peripheral blood mononuclear cells (PBMCs) had been taken after primary (1st) and secondary (2nd) infection or between the acute and convalescent phases. Note to table: in reports in which multiple donors had been used, all percentages were first calculated as % per donor and then averaged over all donors. Hence, some percentages in this table can differ from those in the reports in which the value is reported as the % of experiment rather than per donor. Not all antibodies were characterized; hence, values may be lower than 100%. n.d.: not determined. EDI/DII and DIII refer to the structural domains within the E ectodomain.

Furthermore, since binding of one epitope can enhance or diminish binding of antibodies against other epitopes [60–62], it would be interesting to see whether shifts in these ratios influence neutralization of DENV particles by antibodies against specific epitopes. Based on the tables, we tried to estimate the balance between the various targeted epitopes. For primary convalescent sera, a ratio of approximately 3 E antibodies to 1 prM antibody was found. In secondary convalescent cases, this was near 1 on 1.

Furthermore, the E protein consists of three ectodomains (D): E DI–DIII. In humans, DI and DII are immunodominant domains relative to DIII, as 3-fold more antibodies target DI/III than DIII. However, given the large variability, more studies are required to validate the results.

Although a significant proportion of antibodies target the NS proteins, DNA-vaccine trials suggest that these are not pivotal for neutralization of infection [63,64]. Yet, the NS1 antibodies may aid in clearance of infected cells [65]. Here, we will focus on the antibodies that directly bind to the virus and discuss the clinical relevance of these antibodies.

### PrM Antibodies

We and others showed that prM antibodies are poorly neutralizing and highly enhancing [66–70]. Moreover, infection enhancement was seen over a broad range of concentrations, whereas neutralization occurred in a very narrow range and is incomplete [67–70]. Therefore, prM antibodies have been postulated to contribute primarily to antibody-dependent enhancement of dengue infection and severe disease development. Recent analysis, however, showed that although there is a robust prM response (20%–30%) during acute secondary DENV2 infection, there is no difference in the level of prM antibodies between mild and severe cases [71]. Furthermore, prM antibody levels are increased during secondary, tertiary, and quaternary infections (Table 2, S2 Table, and references therein), whereas severe disease is most often associated with secondary infection [72]. Indeed, subsequent functional analysis did not show a specific correlation between the neutralization/enhancement profile of the sera towards prM-containing particles and the onset of severe disease [71]. This suggests that prM antibodies are not a discriminating factor but act as a cofactor in disease development. Yet, given the weakly neutralizing properties of prM antibodies, it is advisable to avoid the presence of prM in vaccines.

### E Antibodies

Many studies have been done to link neutralization to certain epitopes or structural domains of the E protein (Table 2). Most of the antibodies were found to be directed against dengue EDII fusion loop (FL) (Table 2, S1 Table, and references therein). Furthermore, Lai and colleagues found a correlation between serum EDII FL antibodies and the potency of the serum to neutralize heterotypic DENV [46]. The relevance of these human EDII FL antibodies in protection was further strengthened by elegant tests using prM-E proteins or virus-like particles bearing mutations in the FL [46,73,74].

Based on mouse models, the EDIII was initially considered a major antigen for the induction of serotype-specific neutralizing antibodies [75,76]. Surprisingly, quite low fractions of antibodies targeting EDIII were found during human infection [37,77], and similar low fractions were found after infection with other flaviviruses [78–80]. Moreover, depletion of EDIII-reactive antibodies showed that these are not absolutely required for neutralization [37,78,81,82].

This suggests that the neutralization potency is predominantly facilitated by antibodies against EDI, DII, and the FL. However, and importantly, some monoclonal antibodies could not bind to monomers of E or prM but still bound the whole virion [57,58,68,81,83]. These antibodies may interact with quaternary structures [83–85] and effectively freeze the virus particle as it inhibits changes within the E protein that are required for fusion. An example of such quaternary structure is the EDI/DII hinge region, and recently, antibodies targeting this region were found to be serotype-specific and neutralizing [69,84,85]. Antibodies that bind to viral particles but not to protein monomers are potently neutralizing [58,69,83] but appear to be rare [66]. A recent report, however, showed that near 40% of the isolated monoclonal antibodies (mAbs) bind to quaternary structures [83]. To conclude, we see that the DENV E domains I/II are more immunodominant than the EDIII in terms of induction of antibodies in humans. Importantly, both EDI/II and EDIII antibodies were found to possess a similar neutralization potency [86], and the most neutralizing antibodies against flaviviruses appear to target quaternary structures [78,80,83,86], These findings argue for preservation of quaternary structures in DENV vaccines.

### T Cells

The role of T cells in immunity against dengue infection has been extensively reviewed by others [52,87], and we will briefly discuss recent findings regarding the role of T cells in immunity and pathogenesis. Whereas the CD4^+^ T cell response contributes to protection by instructing B cell responses against the virus [21], the importance of cytotoxic (CD8^+^) T cells for protection is still under debate since low T cell responses are seen during acute stages of DENV infection [36]. After peak viremia, peaks in both T cell response and cytokines are seen [36,88], suggesting that cross-reactive CD8^+^ T cells contribute to pathogenesis rather than protection. Furthermore, during secondary infection, T cells (like B cells) suffer from original antigenic sin [22,36,89]. The cross-reactive T cells during acute secondary infection have an altered cytokine responses consisting of low interferon gamma (IFN-γ) and high tumor necrosis factor alpha (TNF-α) [88,90]. This profile has been associated with severe disease [52]. The phenomenon of original antigenic sin might be less persistent in T cells than in B cells [20], as a recent manuscript showed that multifunctional CD8^+^ T cells can be associated with protection against disease in a Sri Lankan population [22].

Clearly, in naïve individuals, the CYD-TDV vaccine does not induce CD8^+^ T cell responses to the NS proteins of DENV. The participants in the CYD trials, however, had high baseline immunity, implying that T cell responses were already present and potentially boosted by the vaccine [20,39,41]. Thus, we cannot conclude whether or not it is important to include T cell immunity for protection and if this should be induced by a vaccine. Yet, the trials had quite low efficacy results despite high antibody titers. Mouse models indicated that protection requires both B and T cells [91] and that CD8^+^ T cells significantly contribute to disease alleviation, even under conditions of ADE [92]. Thus, CD8^+^ T cells likely contribute to clearance of infection when antibodies have failed to prevent infection. Hence, T cells might be more important for DENV immunity than previously appraised.

## Assays for Vaccine Development

Seroconversion upon vaccination is measured with various assays based on either quantification of DENV-binding antibodies (ELISA) or bioassays measuring neutralization of infection [93]. Currently, the WHO considers the plaque reduction neutralization test (PRNT), which is validated to industrial standards, as the gold standard for DENV [93]. In case of the latter, DENV is mixed with serially diluted sera and added to a monolayer of cells. After incubation, an overlay is placed on top of the cells and plaques develop over time. The neutralization potency of the sera is defined as the dilution that neutralized 50% or 90% of the added virions. For JEV, the correlate of protection is 50% neutralization at a dilution of 1:10 or lower (PRNT_50_ titer of ≥10), and similar correlates of protection have been defined for TBEV and YFV [94]. For DENV, the exact cutoff is unknown but was expected to be similar to the viruses mentioned above.

Based on these criteria, the CYD-TDV trials showed good seroconversion rates, yet for DENV2 a particularly low clinical efficacy was seen (Table 2). This shows that the PRNT assay or its interpretation requires further fine tuning in order to find the true correlate of protection. Many parameters can be adjusted [95–97], such as (I) the cell line, (II) the challenge virus strain, and (III) the defined cutoff for seropositivity. Other parameters include incubation temperature [98,99] and virus source [83].

The current PRNT assay employs the Vero cells, an Fc-receptor (FcR)-negative cell line. FcR-negative cells are inclined toward neutralization, as virus-antibody complexes are only internalized via interaction with FcR. Conversely, FcR-positive cells typically show ADE with poor neutralization [50]. Primary myeloid cells are a natural host cell of DENV and support infection in the absence and presence of antibodies, and they could be an alternative to cell lines [100]. As a start, it would be interesting to investigate if neutralization assays performed with PBMCs of vaccinees gives a better correlate of protection than that of Vero cells. It is unlikely that primary cells will be applied in an industrial setting; yet, the above studies will guide future assay development.

Second, distinct DENV genotypes can give significant shifts in the reported seropositivity, giving e.g. 50% reduction [72]. This is not surprising given the 9% variation within a serotype (Fig 1). More robust correlates of protection against a serotype could be found by including multiple genotypes reflecting the breadth within the serotype.

Third, the threshold chosen for seropositivity was a PRNT_50_ of 10. Yet, the threshold of 50% reduction may not be optimal in terms of variability [97], and different thresholds may be needed according to the serotype [101]. Indeed, in case of the JEV vaccines, the PRNT_50_ values were found to differ between the existing genotypes [102]. The DENV vaccine cohorts now provide excellent opportunities to conduct mathematical studies to find better correlates of protection using more stringent criteria for the neutralization threshold and/or serum dilution.

Overall, there is a poor correlation between the current cutoff for seropositivity (PRNT_50_ ≥10) and clinical efficacy of a DENV vaccine [25,103]. Since Sanofi will continue to monitor the vaccine participants for the next 4 years [19,27,28], the present vaccine trials now offer new prospects for studies to define the best assay and criteria that predict which vaccinees have developed protective immunity. Future studies will also benefit from the lesson of these trials, i.e., that too few participants were bled to allow for thorough correlative analysis between the antibody response and individual protection [28].

## Challenges for Future Dengue Vaccines

In this review, we briefly summarized the outcome of the CYD-TDV vaccine trials. The trials showed us that seroconversion of vaccinees does not necessarily correlate to clinical efficacy against symptomatic disease. This stressed how little we actually know about the human adaptive immune responses towards DENV infection. Most attention had been paid to the human antibody response, and the components thereof have been reviewed above (Table 2 and S1 Table). Based on the Sanofi trials and the reports on the human antibody response, some challenging questions are discussed below.

### Better Responses after Flavivirus Priming?

The CYD-TDV trials reported higher antibody titers in individuals who were flavivirus-positive at baseline than in naïve individuals [20,26,104]. Also, priming apparently gives higher chance on tetravalency [20,26] and better efficacy [27,28]. The better efficacy results in primed individuals suggests that the immune response is different in naïve and primed individuals. In naive individuals, only the DENV antibody response is triggered by CYD-TDV, while in primed individuals, B and T cell responses are boosted, the latter likely through flavivirus-broad conserved epitopes. Yet, the lower antibody levels in flavivirus-naïve individuals could not be compensated for by repeated vaccination [26]. This raises the question of whether the vaccine preferentially expands pre-existing (cross-reactive) immunity and weakly induces de novo immunity. If so, the vaccine may be less beneficial for young children in endemic countries and travelers.

### Absolute Requirement for Tetravalency?

The current dogma is that vaccination should induce serotype-specific antibodies against all four DENV serotypes. Pierson and colleagues suggested that all antibodies that can bind and neutralize DENV can also promote enhancement of infection, irrespective of the epitope [105]. If all antibodies support ADE and neutralization, high titers of cross-reactive antibodies may be sufficient for protection. Yet, a recent study showed that inapparent and apparent dengue cases have similar DENV–immunoglobulin G (IgG) titers but can be distinguished based on whether the sera shows heterotypic neutralizing capacity or not [106]. Future studies should address whether protection of infection depends on the balance of monotypic antibodies and heterotypic antibodies and/or the cumulative titer of all DENV antibodies.

### Why Low Efficacy towards DENV2?

The CYD-TDV showed excellent seroconversion but did not result in high efficacy against symptomatic DENV2. The lack of CD8^+^ T cell responses has been suggested as an option [22]. Recently, there is also growing awareness about the role of the genotype used within the vaccine. Various genotypes of the same serotype can co-currently circulate within endemic areas [107,108]. A mismatch in the genotypes can significantly reduce the affinity of the sera to neutralize infection [72] or may even lead to ADE [7,8]. The low efficacy against DENV2 in the Thai Phase IIb trial was suggested to have occurred because of a mismatch in the vaccine genotype and the circulating genotype [25,109]. If mismatches are indeed important, close surveillance and prediction of the circulating genotypes is crucial. Annual reformulation may be beneficial for protection.

### Vaccine Formulation

The formulation and administration regime of the ideal vaccine is a challenging topic. Subunit vaccines with monomer proteins are safe and can be easily reformulated. However, subunit vaccines also induce antibodies against epitopes that are inaccessible on virus particles due to protein-protein interactions [110] and lack quaternary structures, which are currently the most potent epitopes for neutralization [58,69]. Induction of antibodies against quaternary structures could be facilitated by using whole inactivated viruses, attenuated virus strains, or chimeric viruses.

These three options have their pros and cons. Inactivated vaccines are noninfectious and may induce lower titers of neutralizing antibody compared with vaccines or infection [66,78], likely since different gene expression patterns are induced [23,111]. Lastly, attenuated virus strains mimic the actual pathogen as closely as possible, have the desired quaternary structures, and can induce high antibody titers. Yet, the chimeric vaccine lacks DENV-specific CD8^+^ T cell responses. Moreover, attenuated vaccines can mutate after administration and potentially become virulent, causing health risks, e.g., as seen in polio virus vaccines [112,113]. So far, the results of the Sanofi trials show that the attenuated CYD vaccine is very safe, with no evidence of ADE. Follow-up monitoring of these and future cohorts is important to show that the vaccine is safe over prolonged time periods [19]. The paradox of a DENV vaccine is thus that a vaccine should be sufficiently virulent to induce high antibody titers yet still be attenuated to be safe.

In summary, the recent Phase III trials showed safety and excellent seroconversion [24], although seroconversion did not necessarily imply good efficacy, as shown by DENV2. A major challenge for the future would be to define what assay and criteria predict successful immunization and clinical efficacy. Still, the CYD-TDV offers promise to prevent hospitalization and severe dengue hemorrhagic fever, which is encouraging news. These CYD-TDV trials offer plenty of clues to gain more knowledge about the human response against DENV, the cross-reactivity with and potential cross-protection against flaviviruses, and the interpretation of antibody-based neutralization assays. Knowledge on this will aid future vaccine development against other viruses and pathogens than DENV.

**Key Learning Points**

- Vaccines should preferably induce antibodies against quaternary structures.
- Distinct antibody repertoires are seen for primary and secondary infections.
- The CYD-TDV trials offer possibilities for retrospective analysis to identify correlates of protection.
- To find correlates of protection, further validation and standardization of neutralization assays is required.
- T cells could be more important in DENV immunity than previously appreciated.

**Top Papers in the Field**

- Halstead SB, O'Rourke EJ (1977) Dengue Viruses and Mononuclear Phagocytes. I. Infection Enhancement by Non-neutralizing Antibody. J Exp Med 146: 201–217.

*One of the earliest papers raising awareness on the paradoxical role of antibodies in dengue disease*.

- Capeding MR, Tran NH, Hadinegoro SR, Ismail HI, Chotpitayasunondh T, et al. (2014) Clinical Efficacy and Safety of a Novel Tetravalent Dengue Vaccine in Healthy Children in Asia: A Phase 3, Randomized Observer-Masked Placebo-Controlled Trial. Lancet 384: 1358–1365.
- Villar L, Dayan GH, Arredondo-Garcia JL, Rivera DM, Cunha R, et al. (2014) Efficacy of a Tetravalent Dengue Vaccine in Children in Latin America. N Engl J Med 372: 113–123.
- Sabchareon A, Wallace D, Sirivichayakul C, Limkittikul K, Chanthavanich P, et al. (2012) Protective Efficacy of the Recombinant, Live-Attenuated, CYD Tetravalent Dengue Vaccine in Thai Schoolchildren: A Randomized, Controlled Phase 2b Trial. Lancet 380: 1559–1567.

*In these reports*, *the efficacies of the CYD-TDV vaccines are reported for the first time*, *based on large cohorts in Asia and Latin America*. *Although the efficacy against DENV2 is quite enigmatic*, *the overall efficacy against severe disease and hospitalization offers perspective*.

- de Alwis R, Smith SA, Olivarez NP, Messer WB, Huynh JP, et al. (2012) Identification of Human Neutralizing Antibodies That Bind to Complex Epitopes on Dengue Virions. Proc Natl Acad Sci U S A 109: 7439–7444.

*Here*, *the authors show that potently neutralizing antibodies appear to be directed towards quaternary structures*, *thus providing insight on the requirements of a dengue vaccine*.

- Zellweger RM, Miller R, Eddy WE, White LJ, Johnston RE, et al. (2013) Role of Humoral Versus Cellular Responses Induced by a Protective Dengue Vaccine Candidate. PLoS Pathog 9: e1003723.

*This paper shows the importance of T cells in immunity against dengue virus infections*, *clearly advocating against a focus on antibodies alone*.

- Salje H, Rodriguez-Barraquer I, Rainwater-Lovett K, Nisalak A, Thaisomboonsuk B, et al. (2014) Variability in Dengue Titer Estimates from Plaque Reduction Neutralization Tests Poses a Challenge to Epidemiological Studies and Vaccine Development. PLoS Negl Trop Dis 8: e2952

*The translation from in vitro plaque reduction neutralization assays to in vivo protection has been seriously hampered by the lack of uniformity in the assays and controls*. *With this paper*, *the authors are providing insight on the variance of the assays and definitions of neutralization*. *Moreover*, *clear solutions are suggested for the standardization thereof*.

## Supporting Information

S1 Dataset E amino acid sequences used in the review. The information is given as follows: country of isolation_strain_year of isolation (if known).
(DOCX)

S1 Table An overview of the dengue antibody response in human sera. In this table, the focus is on the development after primary (1st) and secondary (2nd) infection, with the stage of disease at the moment of serum sampling being convalescent (conv.) or unknown. If unknown, only the stage is presented. We grouped the results of primary and secondary infections for individual reports in order to visualize the effects of secondary infection on the antigens targeted and the relative magnitude of antibodies against the epitopes. m.p.i.: months post infection. n.d.: Not determined.
(DOCX)

S2 Table An overview of human monoclonal antibodies derived from immortalized B cells. An overview of human B cell-derived monoclonal antibodies from dengue-infected humans whose PBMCs were taken after primary (1st) or secondary (2nd) infection. The stage of disease was either acute (ac) or convalescent (conv.). Note to table: in reports in which multiple donors had been used, all percentages are first calculated as % per donor and then averaged over all donors. Hence, some percentages can differ from reports in which the value is reported as % of the whole experiment. n.d.: not determined. EDI/DII and DIII refer to the structural domains within the E ectodomain. Reports were selected based on whether they (I) were the first to describe the monoclonal antibodies, (II) screened against several epitopes, and (III) used an unbiased approach to generate the monoclonals.
(DOCX)
