# Supplementary material for: The Complexity of a Dengue Vaccine: A Review of the Human Antibody Response
Source: PLoS Negl Trop Dis. 2015 Jun 11;9(6):e0003749. doi: 10.1371/journal.pntd.0003749 (PMC4465930; doi:10.1371/journal.pntd.0003749)
Supplement: S1 Dataset — The information is given as follows: country of isolation_strain_year of isolation (if known). (DOCX) [file pntd.0003749.s001.docx]

**Flaviviridae**

**Name ID Genotype Reference**

Japanese encephalitis virus [1]

Korea_K94P05_1994 AF045551 I

Japan_Ishikawa_1998 AB051292 I

Cambodia_M859_1967 U70410 I

Thailand_ThCMAr4492_1992 D45362 I

Australia_FU_1995 AF217620 II

Malaysia_CNS138-11_1999 AY184213 II

Japan_JaOArS982_1982 NC_001437 III

Thailand_KPP034-35CT_1982 U03693 III

Nepal_B2524_1985 U70392 III

Thailand_Chiang-Mai_1964 U70393 III

Indonesia_JKT6468_1981 AY184212 IV

Malaysia_Muar_1952 HM596272 V

West Nile Virus [2]

Spain_H-1b_2010 AEK99018 Lineage 1a

USA-New York_NY99-6922_1999 ACJ36231 Lineage 1

Kunjin_MRM61C BAA00176 Lineage 1b

Madagascar_1988 HM147823.1 Lineage 2

Czech_ Rabensburg AY765264.1 Lineage 3

Russia-NW-Caucasus_LEIV-krnd88-190_1998 AY277251.1 Lineage 4

India_strain-804994_1980 DQ256376.1 Lineage 5

[3]

Italy-Piave_2011 AFP50426.1 Lineage 1a

Italy-Livenza-31.1_2012 AFR36922.1 Lineage 1a

Tick-borne Encephalitis Virus

Germany_ K23 CAM82856.1 Central European

strain-263 AAA86739.1 Central European

Finland_ Kumlinge-A52_1952 ADQ00969.1 Central European

strain-Neudoerfl P14336.4 Central European

strain-HYPR Q01299.1 Central European

Germany-CE_2006 ACL97686.1 Central European

Russia, Omsk_2008 AGC25640 Siberian

Russia,Tyumen_2006 ADD11905 Siberian

Russia, Tyumen_2008 AGC25644 Siberian

Russia_ Irkutsk-12_2010 AEQ77280.1 Siberian

Russia_ Cht-22_2002 AEQ77279.1 Siberian

Russia_ Cht-653_1995 AEQ77278.1 Siberian

Russia_ Aina_1963 AEQ77277.1 Siberian

Russia-Ural_Isolate-24_2009 ACZ06556.1 Siberian

Russia_Sofjin_2011 CAA27504 Far Eastern

Russia_ 205 AFV41131.1 Far Eastern

Russia_ Irkutsk-1861_2008 AEQ77276.1 Far Eastern

Russia-Primorskii-Kray_ SofjinKSY_1937 AEP25267.2 Far Eastern

Russia-Ural-Sverdlovsk_ Ekb54_1968 ADN33649.1 Far Eastern

Yellow Fever virus [4]

Angola_Angola71 AY968064.1 Angola

Uganda_strain-SE7445_1964 AAA92704 East/Central Africa

Ethiopia_ETH2777_1961 AY839636.1 East/Central Africa

Kenya_KE93-477 U23575.1 East Africa

Nigeria_1991 U23567.1 West Africa I

Senegal_Dar1279_1965 AAA92700 West Africa II

[5]

Peru_1899-81-B4.1 D14458.1 IIB

Brazil_BeAR646536_2008 AFH35043.1 South America I

Brazil_BeH655417_2002 AFH35044 South America I

**Dengue viruses [6]**

**Name ID Genotype**

Dengue virus 1

Thailand_0008_1981a AY732483 I

Thailand_0081_1982a AY732481 I

Thailand_0323_1991a AY732478 I

Thailand_0336_1991a AY732477 I

Thailand_0097_1994a AY732480 I

Thailand_0488_1994a AY732475 I

Thailand_0049_2001a AY732482 I

Thailand_0102_2001a AY732479 I

Philippines_1974 AF425627 II

Indonesia_A88_1988 AB074761 II

Tahiti_2001 AB111070 II

Indonesia_2002 AB111075 II

Thailand_0442_1980a AY732477 III

Thailand_0673_1980a AY732474 III

[7]

Singapore/S275/1990 33741 IV

Thailand/AHF 82-80/1980 11057 IV

Dengue virus 2

Thailand_0038_1974a DQ181806 Asian I

Thailand_0168_1979 P14337.2 Asian I

Thailand_0498_1984 DQ181804 Asian I

Thailand_0263_1995a DQ181800 Asian I

Thailand_0017_1998a DQ181799 Asian I

Thailand_0055_1999a DQ181798 Asian I

Thailand_0078_2001a DQ181797 Asian I

Thailand_0433_1985a DQ181803 Asian I

Thailand_0026_1988a DQ181802 Asian I

Thailand_New-Guinea-C_1944 P14340.2 Asian II

Thailand_0284_1990a DQ181801 Asian/American

Jamaica_N1409_1983 M20558 Asian/American

Martinique_1998 AF208496 Asian/American

Dominican_DR59_2001 AB122022 Asian/American

Tonga_1974 AY744147 American

Columbia_1986 AY702040 American

Venezuela_2_1987 AF100465 American

Venezuela_131_1992 AF100469 American

Australia_TSV01_1993 AY037116 Cosmopolitan

Indonesia_98900663_1998 AB189122 Cosmopolitan

China_FJ11_1999 AF359579 Cosmopolitan

Indonesia_BA05i_2004 AY858035 Cosmopolitan

Dengue virus 3

Indonesia_1978 AY648961 I

Tahiti_1989 AY744677 I

Thailand_0055_1993 AY676351 II

Thailand_0104/1993 AY676350 II

Thailand_1283_1998 AY676349 II

Thailand_1687_1998 AY676348 II

Thailand_0007_1987 AY676353 II

Thailand_0010_1987 AY676352 II

Martinique_1999 AY099337 III

Sri-Lanka_1266_2000 AY099336 III

Puerto-Rico_1963 AY146762 IV

Puerto-Rico1977 AY146761 IV

Dengue virus 4

Thailand_0087_1977 AY618991 I

Thailand_0348_1991 AY618990 I

Thailand_0485_2001 AY618992 I

China-Guangzhou_strain-B5 AF289029 I

Dominica_1981 AF326573 II

Thailand_0734_2000 AY618993 II

Thailand_0017_1997 AY618989 III

Thailand_0476_1997 AY618988 III

Representatives of each virus genotype were selected based on the literature, and the available envelope protein sequences in the NCBI database. Multiple sequence alignment of the retrieved amino acid sequences was generated by the Clustal X 2.1 program using default parameters. Consensus phylograms were created in SplitsTree 4.13.1^[8]^ by neighbour-joining method with uncorrected p_distance. Scale bar represents the number of (silent) substitutions per site.

References

1.     Solomon T, Ni H, Beasley DW, Ekkelenkamp M, Cardosa MJ, et al. (2003) Origin and evolution of japanese encephalitis virus in southeast asia. J Virol 77(5): 3091-3098.

2.     Bondre VP, Jadi RS, Mishra AC, Yergolkar PN, Arankalle VA. (2007) West nile virus isolates from india: Evidence for a distinct genetic lineage. J Gen Virol 88(Pt 3): 875-884.

3.     Barzon L, Pacenti M, Franchin E, Lavezzo E, Masi G, et al. (2013) Whole genome sequencing and phylogenetic analysis of west nile virus lineage 1 and lineage 2 from human cases of infection, italy, august 2013. Euro Surveill 18(38): 20591.

4.     Mutebi JP, Wang H, Li L, Bryant JE, Barrett AD. (2001) Phylogenetic and evolutionary relationships among yellow fever virus isolates in africa. J Virol 75(15): 6999-7008.

5.     Chang GJ, Cropp BC, Kinney RM, Trent DW, Gubler DJ. (1995) Nucleotide sequence variation of the envelope protein gene identifies two distinct genotypes of yellow fever virus. J Virol 69(9): 5773-5780.

6.     Klungthong C, Putnak R, Mammen MP, Li T, Zhang C. (2008) Molecular genotyping of dengue viruses by phylogenetic analysis of the sequences of individual genes. J Virol Methods 154(1-2): 175-181.

7.     Laille M, Roche C. (2004) Comparison of dengue-1 virus envelope glycoprotein gene sequences from french polynesia. Am J Trop Med Hyg 71(4): 478-484.

8.     Huson DH, Bryant D. (2006) Application of phylogenetic networks in evolutionary studies. Mol Biol Evol 23(2): 254-267.
