# Supplementary material for: The Complexity of a Dengue Vaccine: A Review of the Human Antibody Response
Source: PLoS Negl Trop Dis. 2015 Jun 11;9(6):e0003749. doi: 10.1371/journal.pntd.0003749 (PMC4465930; doi:10.1371/journal.pntd.0003749)
Supplement: S1 Table — In this table, the focus is on the development after primary (1st) and secondary (2nd) infection, with the stage of disease at the moment of serum sampling being convalescent (conv.) or unknown. If unknown, only the stage is presented. We grouped the results of primary and secondary infections for individual reports in order to visualize the effects of secondary infection on the antigens targeted and the relative magnitude of antibodies against the epitopes. m.p.i.: months post infection. n.d.: Not determined. (DOCX) [file pntd.0003749.s002.docx]

Supplemental Table 1: An overview of the dengue antibody response in human sera

| **Stage** | **# sera** | **NS1** | **prM** | **E** | **Values are based on:** | **Reference** |
| --- | --- | --- | --- | --- | --- | --- |
| 1^st^ | 10 | 0% | 30-40% | ≥90% | % patients positive for total Ig against DENV | [1] |
| 2^nd^ | 10 | ≥80% | ≥90% | 100% |  |  |
| 1^st^ | 6 | n.d. | n.d. | 44% DII FL  7.5% DIII | % of DENV-reactive IgG out of the total IgG | [2] |
| 2^nd^ | 6 | n.d. | n.d. | 25% DII FL  4.0% DIII |  |  |
| 1^st^, conv. | 4 | n.d. | 100% – Whole DENV (E-prM) | | % of patients positive for total IgG against DENV | [3] |
| 2^nd^, conv. | 2 | n.d. | 100% – Whole DENV (E-prM) | |  |  |
| 1^st^ | 30 | 100% | n.d. | n.d. | % of patients scoring  DENV-IgG positive by Elisa | [4] |
| 2^nd^ | 10 | 100% | n.d. | n.d. |  |  |
| 1^st^ | 42 | n.d. | 28±1% - Whole DENV (E-prM) | | mean % IgG binding DENV (avidity of individual sera) | [5] |
| 2^nd^ | 18 | n.d. | 61±4% - Whole DENV (E-prM) | |  |  |
| 1^st^ | 19 | 46.6% | 20% | 100% | % Patients with serum-antibodies against DENV | [6] |
| 2^nd^ | 50 | 100% | 100% | 100% |  |  |
| 1^st^, conv | 11 | 81.8% | n.d. | 100% | Patients positive for DENV-reactive B cells | [7] |
| 2^nd^, conv | 17 | 94.1% | n.d. | 100% |  |  |
| 1^st^, 3 m.p.i. | 6 | n.d. | n.d. | 59.0 (35.3) | Average concentration of anti-E IgG antibodies in μg/mL.  (the average % thereof targeting the EDII FL). | [8] |
| 1^st^, 6 m.p.i. | 5 |  |  | 39.6 (31.0) |  |  |
| 1^st^, 12 m.p.i. | 6 |  |  | 35.8 (29.3) |  |  |
| 1^st^, 18 m.p.i. | 4 |  |  | 27.7 (28.7) |  |  |
| 2^nd^ , 3 m.p.i. | 4 | n.d. | n.d. | 833.5 (34.8) |  |  |
| 2^nd^, 6 m.p.i. | 3 |  |  | 810.6 (47.3) |  |  |
| 2^nd^, 12 m.p.i. | 4 |  |  | 364.3 (32.3) |  |  |
| 2^nd^, 18 m.p.i. | 4 |  |  | 329.1 (28.3) |  |  |

References

1.      Valdes K, Alvarez M, Pupo M, Vazquez S, Rodriguez R, et al. (2000) Human dengue antibodies against structural and nonstructural proteins. Clin Diagn Lab Immunol 7(5): 856-857.

2.      Crill WD, Hughes HR, Delorey MJ, Chang GJ. (2009) Humoral immune responses of dengue fever patients using epitope-specific serotype-2 virus-like particle antigens. PLoS One 4(4): e4991.

3.      Wahala WM, Kraus AA, Haymore LB, Accavitti-Loper MA, de Silva AM. (2009) Dengue virus neutralization by human immune sera: Role of envelope protein domain III-reactive antibody. Virology 392(1): 103-113.

4.      Shu PY, Chen LK, Chang SF, Yueh YY, Chow L, et al. (2000) Dengue NS1-specific antibody responses: Isotype distribution and serotyping in patients with dengue fever and dengue hemorrhagic fever. J Med Virol 62(2): 224-232.

5.      Zompi S, Montoya M, Pohl MO, Balmaseda A, Harris E. (2012) Dominant cross-reactive B cell response during secondary acute dengue virus infection in humans. PLoS Negl Trop Dis 6(3): e1568.

6.      Lai CY, Tsai WY, Lin SR, Kao CL, Hu HP, et al. (2008) Antibodies to envelope glycoprotein of dengue virus during the natural course of infection are predominantly cross-reactive and recognize epitopes containing highly conserved residues at the fusion loop of domain II. J Virol 82(13): 6631-6643.

7.      Mathew A, West K, Kalayanarooj S, Gibbons RV, Srikiatkhachorn A, et al. (2011) B-cell responses during primary and secondary dengue virus infections in humans. J Infect Dis 204(10): 1514-1522.

8.      Lai CY, Williams KL, Wu YC, Knight S, Balmaseda A, et al. (2013) Analysis of cross-reactive antibodies recognizing the fusion loop of envelope protein and correlation with neutralizing antibody titers in nicaraguan dengue cases. PLoS Negl Trop Dis 7(9): e2451.
