# Supplementary material for: The Complexity of a Dengue Vaccine: A Review of the Human Antibody Response
Source: PLoS Negl Trop Dis. 2015 Jun 11;9(6):e0003749. doi: 10.1371/journal.pntd.0003749 (PMC4465930; doi:10.1371/journal.pntd.0003749)
Supplement: S2 Table — An overview of human B cell-derived monoclonal antibodies from dengue-infected humans whose PBMCs were taken after primary (1st) or secondary (2nd) infection. The stage of disease was either acute (ac) or convalescent (conv.). Note to table: in reports in which multiple donors had been used, all percentages are first calculated as % per donor and then averaged over all donors. Hence, some percentages can differ from reports in which the value is reported as % of the whole experiment. n.d.: not determined. EDI/DII and DIII refer to the structural domains within the E ectodomain. Reports were selected based on whether they (I) were the first to describe the monoclonal antibodies, (II) screened against several epitopes, and (III) used an unbiased approach to generate the monoclonals. (DOCX) [file pntd.0003749.s003.docx]

Supplemental Table 2: monoclonal antibodies derived from immortalized B cells

| **Reference** | **stage** | **# donors** | **# Abs** | **NS1** | **prM** | **E** | **As % of total E** | |
| --- | --- | --- | --- | --- | --- | --- | --- | --- |
|  |  |  |  |  |  |  | **EDI/DII** | **EDIII** |
| [1] | 1^st^, conv. | 1 | 16 | n.d. | 71.4% | 7.1% |  |  |
| [2] | 1^st^, conv. | 1 | 40 | 7.5% | n.d. | 42.5% |  |  |
| [3] | 1^st^, conv. | 4 (infection) | 37 | n.d. | 35% | 65% | 66.2% | 33.8% |
|  | 1^st^, conv. | 22 (vaccine) | 26 | n.d. | 38% | 62% | 56.5% | 43.5% |
| [4] |  | 12 | 37 | n.d. | 21.6% | 78.4% | 82.8% | 17.2% |
|  | 1^st^ |  |  |  | 14.3% | 85.7% |  |  |
|  | 2^nd^ |  |  |  | 44.4% | 55.6% |  |  |
| [5] | 1^st^, conv. | 3 | 49 | 8.0% | 5.7% | 80.5% | 72.6% | 27.4% |
|  | 2^nd^, conv. | 2 | 29 | 0.0% | 2.6% | 94.8% | 75.0% | 25.0% |
| [6] | 2^nd^, ac. | 2 | 26 | n.d. | n.d. | 92.3% |  | 0% |
| [7] | 2^nd^ ac. | 4 | 121 | 3.3% | 6.6% | 81.8% |  |  |
|  | 2^nd^, conv. | 5 | 15 | 53.3% | 13.3% | 13.3% |  |  |
| [8] | 2^nd^, conv. | 7 | 301 | 32.1% | 40% | 27.4% |  |  |

References

1.      de Alwis R, Beltramello M, Messer WB, Sukupolvi-Petty S, Wahala WM, et al. (2011) In-depth analysis of the antibody response of individuals exposed to primary dengue virus infection. PLoS Negl Trop Dis 5(6): e1188.

2.      Friberg H, Jaiswal S, West K, O'Ketch M, Rothman AL, et al. (2012) Analysis of human monoclonal antibodies generated by dengue virus-specific memory B cells. Viral Immunol 25(5): 348-359.

3.      Smith SA, de Alwis R, Kose N, Durbin AP, Whitehead SS, et al. (2013) Human monoclonal antibodies derived from memory B cells following live attenuated dengue virus vaccination or natural infection exhibit similar characteristics. J Infect Dis 207(12): 1898-1908.

4.      Smith SA, Zhou Y, Olivarez NP, Broadwater AH, de Silva AM, et al. (2012) Persistence of circulating memory B cell clones with potential for dengue virus disease enhancement for decades following infection. J Virol 86(5): 2665-2675.

5.      Beltramello M, Williams KL, Simmons CP, Macagno A, Simonelli L, et al. (2010) The human immune response to dengue virus is dominated by highly cross-reactive antibodies endowed with neutralizing and enhancing activity. Cell Host Microbe 8(3): 271-283.

6.      Xu M, Hadinoto V, Appanna R, Joensson K, Toh YX, et al. (2012) Plasmablasts generated during repeated dengue infection are virus glycoprotein-specific and bind to multiple virus serotypes. J Immunol 189(12): 5877-5885.

7.      Setthapramote C, Sasaki T, Puiprom O, Limkittikul K, Pitaksajjakul P, et al. (2012) Human monoclonal antibodies to neutralize all dengue virus serotypes using lymphocytes from patients at acute phase of the secondary infection. Biochem Biophys Res Commun 423(4): 867-872.

8.      Dejnirattisai W, Jumnainsong A, Onsirisakul N, Fitton P, Vasanawathana S, et al. (2010) Cross-reacting antibodies enhance dengue virus infection in humans. Science 328(5979): 745-748.
